# Orienteering and adolescent depressive and anxiety symptoms: indirect associations via psychological resilience and moderation by ADHD symptoms

**DOI:** 10.3389/fpsyg.2026.1843028

**Published:** 2026-07-01

**Authors:** Mei Jiang, Longjiang Chen, Mingru Lei, Zhengyang Mei

**Affiliations:** 1 College of Physical Education, Chengdu University of Technology, Chengdu, China; 2 School of Physical Education, Yunnan Normal University, Kunming, China; 3 School of Education, Yunnan Normal University, Kunming, China; 4 School of Physical Education, Southwest University, Chongqing, China

**Keywords:** ADHD symptoms, adolescent mental health, anxiety, depression, orienteering, psychological resilience

## Abstract

**Objective:**

To examine whether participation in orienteering is associated with adolescents’ depression and anxiety, and to examine whether the association between orienteering participation and depressive or anxiety symptoms was statistically linked to resilience, and whether ADHD symptoms moderated this indirect association.

**Methods:**

A cross-sectional survey was conducted among adolescents aged 10–19 years in Sichuan, China (*N* = 1,154). Participants reported orienteering participation, resilience, ADHD symptoms, depression, and anxiety. Moderated mediation was tested using PROCESS, and predictive models (ridge regression and XGBoost) with SHAP values were used as a robustness check.

**Results:**

Orienteering participation was positively associated with resilience, and resilience was negatively associated with depression and anxiety. The direct associations between orienteering participation and depression/anxiety were not significant; however, resilience showed significant indirect associations linking orienteering participation to lower depression and anxiety. ADHD symptoms moderated the second stage of the mediation, such that the protective indirect association via resilience was stronger at higher levels of ADHD symptoms (index of moderated mediation = −0.02 for both outcomes). Machine-learning models yielded moderate predictive performance (holdout *R*^2^ ≈ 0.35 for depression and 0.29 for anxiety), with ADHD symptoms, resilience, and their interaction among the most influential predictors.

**Conclusion:**

Orienteering participation was associated with fewer internalizing problems, and this association was statistically reflected in higher resilience. This indirect association was more pronounced among adolescents with elevated ADHD symptoms.

## 1 Introduction

Adolescence is a key period of rapid biopsychosocial reorganization, during which heightened emotional reactivity, rising peer and academic stress, and still-maturing self-regulatory systems increase the risk of internalizing problems such as depression and anxiety (Kim et al., 2025; Klinge et al., 2023). Such psychological distress not only impairs adolescents’ academic functioning, social adjustment, and quality of life but may also exert lasting effects on later development (Orban et al., 2024; Swan et al., 2018). Therefore, it is important to identify mental health–promoting approaches that can be scaled in school or community settings and have high feasibility and acceptability. Physical activity is generally believed to be an approach that can help promote the improvement of mental health; However, with increasing evidence supporting the psychological benefit differences among various physical activities; For example, those that require higher levels of “cognitive engagement” have been shown to have relatively stronger links with the underlying mechanism of emotional regulation and stress coping (Świda et al., 2025). In terms of orienteering, which is an integration of physical exertion with ongoing navigation-related decisions and has the potential to provide a specific path for exploring how physical activities impact mental state (Waddington et al., 2024). However, systematic evidence on orienteering and adolescent depression and anxiety remains limited, and it is still unclear through which psychological resource pathways this association operates and for which adolescents it is more pronounced.

### 1.1 Orienteering and adolescent depression and anxiety

Orienteering (OR) is a recreational activity involving navigation by using maps and a compass to reach a series of control points (often abbreviated to CPs) in the shortest possible time or with other specified times (such as the fastest time), routes, and combinations (Waddington et al., 2024). Unlike the repetition of physical effort-dominated exercise mode, orienteering integrates cognitive assessment into one task for a longer time with less physical exertion: Participants need to maintain focus continuously under stress in outdoor environment conditions; They are required to distinguish and sort out environmental information quickly during decision-making at any moment; And after making decisions, it should be corrected timely and avoid losing the goal. With a dual-task composition of cognition and movement, there are also potential beneficial effects on the improvement of psychological state under this kind of exercise method, in addition to physical fitness, such as enhancing cognitive engagement and self-regulation ability (Araujo et al., 2019; Biehl-Printes et al., 2024). The above findings also show that orienteering has an association with improved emotion regulation (Yen et al., 2025). Moreover, as long as people have a continuous need to switch strategies or monitor errors in orienteering, it is likely that the relationship between strategy-switching behavior and emotion regulation, stress reduction methods, and other aspects will be strengthened; And because most of them take place outdoors under natural light conditions, such settings can help restore one’s state and reduce negative effects. Therefore, orienteering may be particularly relevant to adolescent mental health because it combines physical exertion, environmental exploration, sustained attention, and real-time problem solving, all of which are closely related to emotional regulation and stress adaptation.

### 1.2 Resilience as a mediating mechanism

To explain how orienteering may relate to depression and anxiety, this study treats psychological resilience as a key mediator. Psychological Resilience (RES) refers to the ability of individuals to adapt positively or develop effectively after encountering major threats and difficulties (Wright and Masten, 2005). Conservation of Resources (COR) theory suggests that RES can reduce stress by promoting adaptive behavior through its coping mechanism, but it is found that depression and anxiety are often associated with a lack of resources and cumulative pressure according to this theory (Hobfoll, 1989). Meta-analytic research has also shown that psychological resilience may be negatively related to some adverse psychological conditions such as depression and anxiety (Ghanei Gheshlagh et al., 2017). Orienteering provides a naturalistic context for building resilience-related resources: under concurrent time and physical demands, participants must continually set goals, verify position, and monitor actions; when route judgments go awry or they become disoriented, they must rapidly reorient and adjust strategy to resume task execution. This process reflects a dynamic psychological cycle of “monitoring–adjusting–re-engaging” (Baumeister and Vohs, 2007; Hobfoll, 1989), and requires the integration of core resilience components such as cognitive flexibility, self-regulation, and emotion control (Bonanno, 2004). Accordingly, orienteering is not merely physical training but an ecological dual-task activity characterized by cognitive challenge and stress adaptation (Batista et al., 2020); through repeated goal–feedback–adjustment cycles, individuals may strengthen resource conservation and recovery processes, thereby facilitating the development and consolidation of psychological resilience (Hobfoll, 1989). Taken together, these theoretical and empirical considerations suggest that resilience may be a key psychological factor statistically linking orienteering participation with lower depressive and anxiety symptoms.

### 1.3 The moderating role of attention-deficit/hyperactivity disorder symptoms

Notably, the psychological benefits of exercise are not equal for all adolescents (Fu et al., 2025). Such heterogeneity is likely to be related to the extent that the activity requires the use of executive functions, such as sustained attention, plan formulation and execution, error detection and correction, etc., as well as individual differences in these abilities. Orienteering requires strong reliance on these cognitive activities during participation (Baumeister and Vohs, 2007), and therefore, individual characteristics related to executive functions are more prone to explain the differences in psychological benefits of orienteering. In this study, attention-deficit/hyperactivity disorder symptoms (ADHD-Sx) are used to index the level or severity of inattention and hyperactive/impulsive symptoms, which are considered a continuous dimension rather than a diagnostic category. Difficulty in sustaining attention and controlling impulses reflected in this symptom dimension may reduce involvement and self-regulation during participation, thus affecting the quality of subjective experience and further impacting the strength of the path by which psychological resources are converted into emotional benefits (Carpenter et al., 2019). Adolescents with higher ADHD-Sx often have greater self-regulatory demands and a higher risk of emotional lability (Shaw et al., 2014), and psychological resilience, as a kind of capacity for adaptation, coping with stress and regulating negative emotions, may be an essential protective resource in this context. In terms of the risk-protective/resource-compensation effect, the impact of protective factors will be enhanced under increased risk (English et al., 2018; Fergus and Zimmerman, 2005). That is to say, when the ADHD-Sx score is higher, the negative correlation between resilience and depression/anxiety is more pronounced; while in people with low ADHD-Sx, the baseline risk of negative emotions is lower, so the marginal protective effect of resilience on outcomes may be relatively small. Therefore, by introducing ADHD-Sx as a moderator, it can be explained that adolescents may show different degrees of emotional improvement under similar levels of resilience; thus, more precisely defining the target group and boundary conditions for orienteering-based interventions. Because orienteering requires sustained attention, inhibitory control, planning, and error correction, adolescents with higher ADHD-Sx may show different patterns in how resilience is associated with internalizing symptoms in this cognitively demanding sport context.

### 1.4 Current study

Although a substantial body of research supports the association between physical activity and adolescent mental health, existing evidence on orienteering—an activity that combines physical load with sustained cognitive–executive demands—has yet to address three key questions. First, is the association between orienteering participation and internalizing problems such as depression and anxiety reliably present? Second, can this association be explained by identifiable psychological mechanisms, particularly whether psychological resilience serves as a key mediator between orienteering and internalizing problems? More importantly, psychological resources do not translate into emotional benefits with equal strength across individuals: do risk-context factors (e.g., ADHD-Sx) alter the protective role of resilience for depression and anxiety, thereby producing individual differences in the indirect association of orienteering on internalizing problems via resilience? In response to these questions, the theoretical contribution of this study is to integrate orienteering, resilience, and internalizing problems into a single framework that also incorporates differential protective effects under risk contexts, thereby more precisely specifying the conditions and boundaries under which orienteering yields psychological benefits; its practical contribution is to provide risk-stratified intervention implications for school-based physical education and outdoor programs, and to clarify the key protective role of resilience among adolescents with elevated ADHD-Sx.

To strengthen evidential completeness and testability, this study adopts an analytic strategy that combines explanatory modeling with predictive modeling: on the explanatory side, mediation and moderation pathways are tested within a regression/PROCESS framework; on the predictive side, 5-fold cross-validated comparisons using Ridge regression and XGBoost are conducted, and SHAP is used to identify key predictive features and their relative contributions, to assess robustness across modeling approaches and explore potential nonlinearity. Importantly, the predictive analyses are intended to complement and cross-validate the mechanistic conclusions rather than replace theory-driven inference. Based on the theoretical model, this study proposes the following hypotheses: H1: Orienteering participation is significantly associated with adolescents’ levels of depression and anxiety. H2: Psychological resilience shows an indirect association in the relationship between orienteering participation and depression or anxiety. H3: ADHD-Sx moderates the path between resilience and depression/anxiety, such that the indirect association of orienteering on depression/anxiety via resilience differs across levels of attention-deficit symptoms.

## 2 Methods

### 2.1 Participants

This study collected anonymous online survey data via the Wenjuanxing platform. The questionnaire was distributed and returned through social networking channels. Participants were adolescents from Sichuan, China, with some prior orienteering experience; a total of 1,362 questionnaires were returned. During data collection, a survey layout technique was used to reduce response carryover and potential common method bias: first, different scales were presented on separate pages; second, a mandatory 15-s delay was imposed before participants could proceed to the next page to mitigate inertia in consecutive responding. To minimize the impact of careless or random responding, invalid questionnaires were excluded based on predefined data-quality criteria, including: (1) completion time < 1 min; (2) an obvious straightlining pattern in scale items (the same option selected more than six times consecutively); and (3) clearly abnormal key information or evident random/fill-in errors. After exclusions, the final valid sample was *N* = 1,154. Participants ranged in age from 10 to 19 years (*M* = 14.86, SD = 2.02). The sample included 694 males (60.14%) and 460 females (39.86%). Duration of participation in orienteering ranged from 1 to 72 months (*M* = 9.30, SD = 11.39).

### 2.2 Research tools

Orienteering involvement was assessed using the Physical Activity Rating Scale, revised by Liang (1994), which measures adolescents’ frequency, intensity, and duration of orienteering participation over the past month. The scale includes three items rated on a 1–5 scale. The total physical activity score was computed as Frequency × Intensity × (Duration − 1), yielding scores from 0 to 100; scores ≤19 indicate low activity, 20–42 indicate moderate activity, and ≥43 indicate high activity, with higher scores reflecting greater activity volume. In this study, Cronbach’s *α* for the Physical Activity Rating Scale was 0.61, and the KMO value was 0.59. Although this reliability coefficient was lower than those of the psychological symptom scales used in the present study, it exceeded the minimum criterion of 0.60 that some scholars regard as acceptable in exploratory research, particularly for brief instruments. More importantly, this scale assesses activity frequency, exercise intensity, and exercise duration as complementary components of overall activity volume rather than as highly homogeneous or interchangeable Likert-type indicators. Therefore, conventional internal-consistency indices may be less suitable for evaluating this behavioral composite measure, because frequency, intensity, and duration are not necessarily expected to show strong inter-item correlations.

Psychological resilience was measured with the 10-item unidimensional Connor–Davidson Resilience Scale (CD-RISC-10), revised by Campbell-Sills and Stein (2007), which assesses the ability to cope with adversity and stress and to recover from setbacks. Items are rated on a 5-point scale from 0 (not true at all) to 4 (true nearly all of the time). Example items include “able to adapt to change,” “able to deal with whatever comes,” and “able to handle unpleasant feelings.” Higher total scores indicate greater resilience. In this study, Cronbach’s *α* was 0.97, and the KMO was 0.96.

Depressive symptoms were assessed using the 10-item version of the Center for Epidemiologic Studies Depression Scale–Revised for adolescents (CESDR-10), adapted by Haroz et al. (2014), a unidimensional measure. Each item uses a 5-point frequency scale to assess symptom frequency over a recent period (e.g., 0 = “rarely or none of the time in the past week/less than 1 day,” 1 = “1–2 days,” 2 = “3–4 days,” 3 = “5–7 days,” 4 = “nearly every day in the past two weeks”). Example items include “I felt sad” and “I felt irritable.” Higher scores indicate greater depressive symptom severity. In this study, Cronbach’s *α* was 0.91, and the KMO was 0.93.

Anxiety was measured using the 6-item Generalized Anxiety Disorder scale (GAD-6) adapted by Todorović et al. (2023), which assesses the frequency of anxiety symptoms over a recent period. Items are rated on a frequency scale from 0 (“not at all”) to 2 (“nearly every day”). The scale has a unidimensional structure, with higher scores indicating greater anxiety severity. Example items include “I felt nervous, anxious, or on edge” and “I found it hard to relax.” In this study, Cronbach’s *α* was 0.90, and the KMO was 0.90.

ADHD symptoms (ADHD-Sx) were assessed using the 6-item Adult ADHD Self-Report Scale v1.1 Screener (ASRS v1.1 Screener) used and psychometrically evaluated by Green et al. (2019), and measures the frequency of ADHD-related symptoms over the past 6 months. Items are rated on a 5-point frequency scale (1 = “never,” 2 = “rarely,” 3 = “sometimes,” 4 = “often,” 5 = “very often”), and the scale is unidimensional. Example items include “When a project is near completion, I have difficulty getting the final details done” and “I fidget or squirm when I have to sit for a long time.” Higher scores indicate higher levels of ADHD-Sx. In this study, Cronbach’s α was 0.90, and the KMO was 0.88.

### 2.3 Data processing

In this study, using SPSS 27.0 for descriptive statistics, correlation analysis, and reliability test of the main variable’s data; Utilizing PROCESS macro (Version 4.2) to examine the mediating and moderating effects.

### 2.4 Machine-learning predictive modeling (XGBoost) and model interpretability analysis

The machine-learning analysis was used as a complementary predictive analysis rather than as a substitute for the theory-driven moderated mediation model. PROCESS was used to test the hypothesized indirect and conditional associations, whereas XGBoost and SHAP were used to examine whether the theoretically specified variables also contributed meaningfully to prediction. To evaluate the predictive performance and robustness of the theory-driven set of variables for depressive and anxiety symptoms, this study employed machine-learning predictive modeling and reported results from both cross-validation and an independent holdout test set. Prior to analysis, the sample was randomly split into a development set and a final holdout test set. The holdout test set comprised 20% of the sample and was not used for any training, hyperparameter tuning, or model comparison; it was reserved solely for a one-time evaluation of out-of-sample generalization. The remaining 80% of the sample served as the development set for model development and performance estimation. For preprocessing, to align the scales of each variable so that they are more comparable across different types, enhance the numerical stability required by the model for handling interaction terms and regularized regression, and standardize non-gender variables. Therefore, RMSE and MAE have been conventionally used to describe the prediction error in standard deviation units. To maintain consistency between the explanatory and predictive analyses, the feature set included the key variables specified in the moderated mediation model: orienteering participation, psychological resilience, ADHD-Sx, the Resilience × ADHD-Sx interaction, and covariates including gender, age, and duration of orienteering experience.

In the training and validation sets during model selection, a split ratio of roughly 80:20 was adopted to perform fivefold cross-validation; i.e., approximately 80 percent of the samples were selected as training samples while the remaining 20 percent served primarily for performance evaluation. The same partitioning of folds, ridge regression, and gradient-boosted tree regression models were fitted for evaluation to ensure that they could be compared. Ridge regression was used as a regularized linear benchmark model, which estimates additive associations while reducing overfitting through coefficient shrinkage. In contrast, XGBoost is a gradient-boosted decision-tree model that can capture nonlinear patterns and higher-order feature interactions, thereby providing a more flexible comparison with the linear model. For ridge regression, the penalty parameter was selected through cross-validation in each outer training fold; For gradient boosting, the optimal number of boosting iterations was determined in combination with early stopping while performing cross-validation. Performance of the model was evaluated on each outer test fold using *R*^2^, RMSE, and MAE, and the mean and standard deviation of cross-validation were provided to assess its stability. Subsequently, the final model was trained on the full development set and evaluated once on the pre-specified holdout test set to report final generalization performance. To reduce optimistic bias that can arise when interpreting models on training data, feature-importance interpretation followed a test-only strategy: within the 5-fold cross-validation framework on the development set, SHAP values were computed for each fold using the model trained on that fold’s training data to explain predictions on that fold’s test set only. SHAP values from all test folds were then aggregated to derive overall interpretability results.

## 3 Results

### 3.1 Common method bias

Common method bias was assessed using Harman’s single-factor test. The results showed five factors with eigenvalues greater than 1, and the first unrotated factor accounted for 34.878% of the total variance, which is below the 40% cutoff. Therefore, common method bias was not considered a concern in this study.

### 3.2 Descriptive statistics and correlation analysis

Means, standard deviations, and correlation coefficients for the main variables are presented in Table 1. Correlation analyses indicated that orienteering participation was significantly positively correlated with psychological resilience (*r* = 0.14, *p* < 0.001), and significantly negatively correlated with ADHD symptoms (*r* = −0.08, *p* < 0.01), depressive symptoms (*r* = −0.11, p < 0.01), and anxiety symptoms (*r* = −0.10, *p* < 0.01). Psychological resilience was significantly negatively correlated with both depression (*r* = −0.28, *p* < 0.001) and anxiety (*r* = −0.23, *p* < 0.001), and also significantly negatively correlated with ADHD-Sx (*r* = −0.16, *p* < 0.001). ADHD-Sx was significantly positively correlated with depression (*r* = 0.63, *p* < 0.01) and anxiety (*r* = 0.65, *p* < 0.001), and depression and anxiety were strongly positively correlated (*r* = 0.80, *p* < 0.001). In addition, gender was significantly positively correlated with depression (*r* = 0.18, *p* < 0.001) and anxiety (*r* = 0.19, *p* < 0.001). Duration of exposure to orienteering was significantly positively correlated with orienteering participation (*r* = 0.09, *p* < 0.001) and weakly negatively correlated with anxiety (*r* = −0.06, *p* < 0.05).

**Table 1:** Descriptive statistics and correlations among study variables.

Variables	*M*	SD	1	2	3	4	5	6	7	8
1. Age	16.12	23.86	1							
2. SEX	1.40	0.49	0.02	1						
3. EXP	9.30	11.39	0.04	−0.2	1					
4. OR	21.25	18.01	−0.01	−0.23	0.09**	1				
5. DEP	4.44	5.84	−0.02	0.18**	−0.08	−0.1	1			
6. ANX	1.81	2.45	−0.01	0.19**	−0.06*	−0.10**	0.80**	1		
7. ADHD-Sx	4.50	4.65	−0.02	0.09**	−0.01	−0.08**	0.63**	0.65**	1	
8. RES	23.08	11.00	0.01	−0.03	0.01	0.14**	−0.28**	−0.23**	−0.16**	1

* Correlation is significant at the 0.05 level (two-tailed). ** Correlation is significant at the 0.01 level (two-tailed). *** Correlation is significant at the 0.001 level (two-tailed). Age = age (years), SEX = sex (0 = male, 1 = female), EXP = orienteering experience (months), OR = orienteering participation level (past month), DEP = depressive symptoms, ANX = anxiety symptoms, ADHD-Sx = attention-deficit/hyperactivity disorder symptom level (continuous), RES = psychological resilience (same hereafter).

### 3.3 Mediation analysis

Using the PROCESS macro (Model 4) to examine whether psychological resilience was statistically involved in the indirect association between orienteering participation and negative emotions. Parameter estimates were obtained using bootstrapping (5,000 resamples), and statistical significance was determined by whether the 95% confidence interval (95% CI) of the estimate included zero. Age, sex, and orienteering experience (months) were included as covariates.

The Results show that the overall association of participation in orienteering on depression is significantly negative (*c* = −0.02, *p* < 0.05); The total association of participation in orienteering on anxiety is also very high (−0.01, *p* < 0.05). The mediating model shows that orienteering participation is significantly and positively correlated with resilience (*β* = 0.15, *p* < 0.01), and resilience is significantly and negatively associated with depression (*β* = −0.27, *p* < 0.01) and anxiety (*β* = −0.22, *p* < 0.01). After the inclusion of resilience, the direct association between orienteering participation and depression was not significant (*c*′ = −0.01, 95% CI [−0.025, 0.012], *p* = 0.48), nor was the direct association between orienteering participation and anxiety (*c*′ = 0.00, 95% CI [−0.012, 0.004], *p* = 0.36). Bootstrap further showed that the indirect association between orienteering participation and depression via resilience was significant (ab = −0.013, 95% CI [−0.020, −0.006]), and the indirect association for anxiety was also significant, although small in magnitude (ab = −0.004, 95% CI [−0.007, −0.002]). Based on the above data, The results were consistent with an indirect association through resilience. The association between orienteering participation and depression/anxiety was mainly reflected in the statistical indirect association via resilience. (See Table 2).

**Table 2:** Indirect association estimates involving psychological resilience in the associations between orienteering participation and depression/anxiety.

Variables	Equation 1 (RES)		Equation 2 (DEP)		Equation 3 (ANX)	
	*β*	*t*	*β*	*t*	*β*	*t*
OR	0.15	4.86***	−0.02	−0.70	−0.03	−0.93
RES			−0.27	−9.60***	−0.22	−7.77*
Age	0.01	0.45	−0.02	−0.60	−0.02	−0.51
SEX	0.01	0.21	0.16	5.40***	0.17	5.75*
EXP	0.00	−0.03	−0.04	−1.53	−0.02	−0.78
*R* ^2^	0.02		0.11		0.09	
*F*	6.18***		27.83***		21.84***	

### 3.4 Moderated mediation analysis

**Figure 1:**
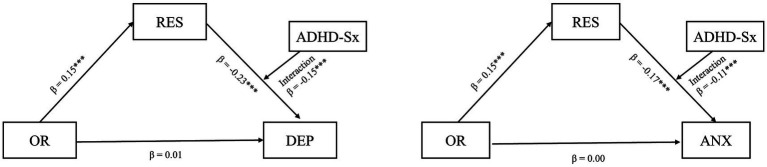
Standardized path coefficients of the moderated mediation models for depressive and anxiety symptoms.

A moderated mediation model was tested using SPSS PROCESS Macro (Model 14). Using 5,000 bootstrap resamples, and deeming the effect significant when the 95% confidence interval (95% CI) did not contain zero. Sex, age, and orienteering experience (months) were included as covariates. The results showed that orienteering participation significantly and positively predicted psychological resilience (*β* = 0.15, *t* = 4.86, *p* < 0.001, 95% CI [0.09, 0.21]). The standardized path coefficients of the moderated mediation models are shown in Figure 1, and the detailed regression results are presented in Table 3. When depression was the result, resilience significantly and negatively predicted depression (*β* = −0.24, *t* = −9.61, *p* < 0.001, 95% CI [−0.28, −0.19]), and the Resilience × ADHD-Sx interaction also significantly predicted depression (*β* = −0.15, *t* = −6.38, *p* < 0.001, 95% CI [−0.20, −0.10]). When anxiety was the result, resilience significantly and negatively predicted anxiety (*β* = −0.17, *t* = −6.77, *p* < 0.001, 95% CI [−0.21, −0.12]), and the Resilience × ADHD-Sx interaction significantly predicted anxiety (*β* = −0.11, *t* = −4.65, *p* < 0.001, 95% CI [−0.16, −0.06]). These results suggest that ADHD-Sx moderated the association between resilience and depression/anxiety, that is, the second-stage path of the mediation model.

**Table 3:** Regression results for moderated mediation.

Variables	Equation 1 (RES)		Equation 2 (DEP)		Equation 3 (ANX)	
	*β*	*t*	*β*	*t*	*β*	*t*
OR	0.15	4.86***	0.01	0.31	0.00	−0.02
RES			−0.23	−9.61***	−0.17	−6.77***
ADHD-Sx			0.55	24.13***	0.59	25.70***
RES × ADHD-Sx			−0.15	−6.38***	−0.11	−4.65***
SEX	0.01	0.21	0.20	4.18***	0.22	4.80***
Age	0.01	0.45	0.00	−0.01	0.00	0.11
EXP	0.00	−0.04	−0.04	−1.97*	−0.02	−1.11

Further moderation mediation tests showed that the direct association of orienteering participation on both depression and anxiety were not significant; However, the conditional indirect associations of orienteering participation on depression/anxiety through resilience were significant at different levels of ADHD-Sx and strengthened as ADHD-Sx increased. The indices of moderated mediation were also all statistically significant: the index for depression was −0.02 (95% CI [−0.046, −0.007]); The index for anxiety was −0.02 (95% CI [−0.035, −0.004]). Since both confidence intervals excluded zero, the moderated mediation effect is supported. To determine the specific form of this moderation, slope analysis was performed at the low (−1 SD), average, and high (+1 SD) levels of ADHD-Sx. For depression, resilience significantly predicted lower depression at low ADHD-Sx (simple slope = −0.09, *t* = −3.47, *p* < 0.001), with a stronger association at the mean level (simple slope = −0.24, *t* = −9.61, *p* < 0.001), and an even stronger association at high ADHD-Sx (simple slope = −0.39, *t* = −9.55, *p* < 0.001). For anxiety, resilience also significantly predicted lower anxiety at low ADHD-Sx (simple slope = −0.06, *t* = −2.31, *p* = 0.02), with a stronger association at the mean level (simple slope = −0.17, *t* = −6.77, *p* < 0.001), and a further strengthened association at high ADHD-Sx (simple slope = −0.28, *t* = −6.82, *p* < 0.001). In summary, although higher ADHD-Sx was associated with more severe depression and anxiety, the negative association of resilience was more pronounced in adolescents with higher ADHD-Sx, thereby resulting in a greater indirect association of orienteering participation on depression and anxiety via resilience.

### 3.5 Machine-learning prediction and feature contribution analysis

Based on the 5-fold cross-validation results in the development set, the two models showed broadly comparable predictive performance for both depression and anxiety, with limited between-fold variability, indicating good stability across different data splits (see Table 4). For depression, ridge regression and XGBoost performed similarly in cross-validation (*R*^2^ = 0.440 ± 0.080 vs. 0.426 ± 0.099; RMSE = 0.735 ± 0.090 vs. 0.744 ± 0.106; MAE = 0.497 ± 0.060 vs. 0.502 ± 0.067). For anxiety, ridge regression performed slightly better than XGBoost in cross-validation (*R*^2^ = 0.454 ± 0.077 vs. 0.426 ± 0.082; RMSE = 0.742 ± 0.046 vs. 0.762 ± 0.060; MAE = 0.540 ± 0.029 vs. 0.547 ± 0.031), although the differences were small. In the final holdout test set, performance declined for both outcomes relative to cross-validation: for depression, *R*^2^ was 0.345 (Ridge) and 0.356 (XGBoost), RMSE was 0.824 and 0.817, and MAE was 0.567 and 0.555, respectively; for anxiety, *R*^2^ was 0.294 and 0.298, RMSE was 0.799 and 0.797, and MAE was 0.583 and 0.582, respectively. Overall, given the current feature set and sample conditions, the more complex non-linear ensemble model did not show a significant superiority over the linear baseline in this study; therefore, differences between them were relatively small on the independent test set. These results suggest that, within the current set of variables, the linear and nonlinear models yielded broadly comparable predictive performance, and the more complex XGBoost model did not provide clear incremental improvement over ridge regression. and thus, the sensitivity of the selection outcome to different algorithms is not strong.

**Table 4:** Model performance on the development set (5-fold CV) and the final holdout test set (20%).

Outcome	Model	Training set (*n*)	Test set (*n*)	5-fold CV RMSE	5-fold CV MAE	5-fold CV *R*^2^	Holdout RMSE	Holdout MAE	Holdout *R*^2^
DEP	Ridge	924	230	0.735 ± 0.090	0.497 ± 0.060	0.440 ± 0.080	0.824	0.567	0.345
ANX	XGBoost	924	230	0.744 ± 0.106	0.502 ± 0.067	0.426 ± 0.099	0.817	0.555	0.356
DEP	Ridge	924	230	0.742 ± 0.046	0.540 ± 0.029	0.454 ± 0.077	0.799	0.583	0.294
ANX	XGBoost	924	230	0.762 ± 0.060	0.547 ± 0.031	0.426 ± 0.082	0.797	0.582	0.298

**Figure 2:**
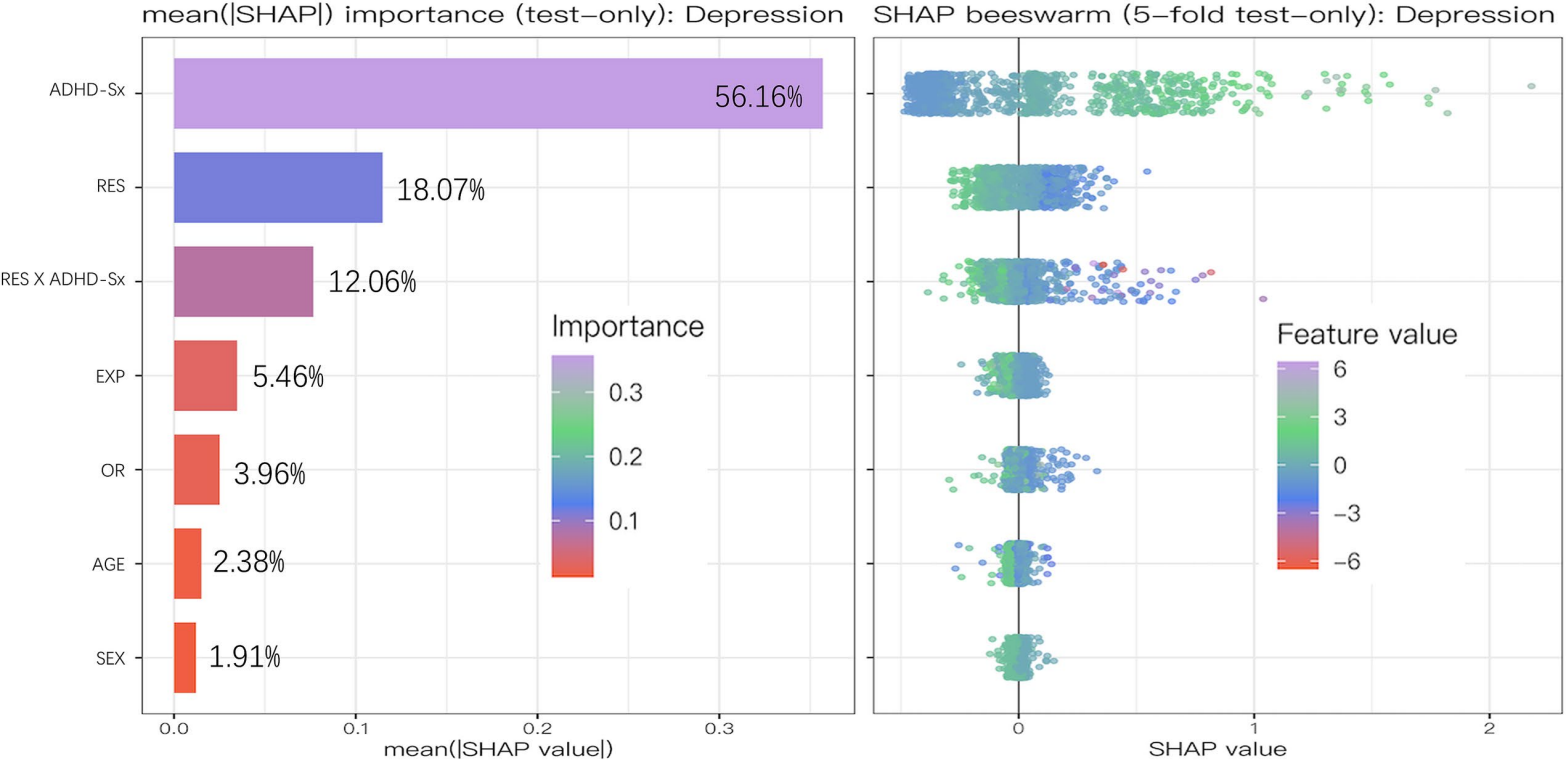
Feature importance and SHAP explanations for the XGBoost model predicting depression.

**Figure 3:**
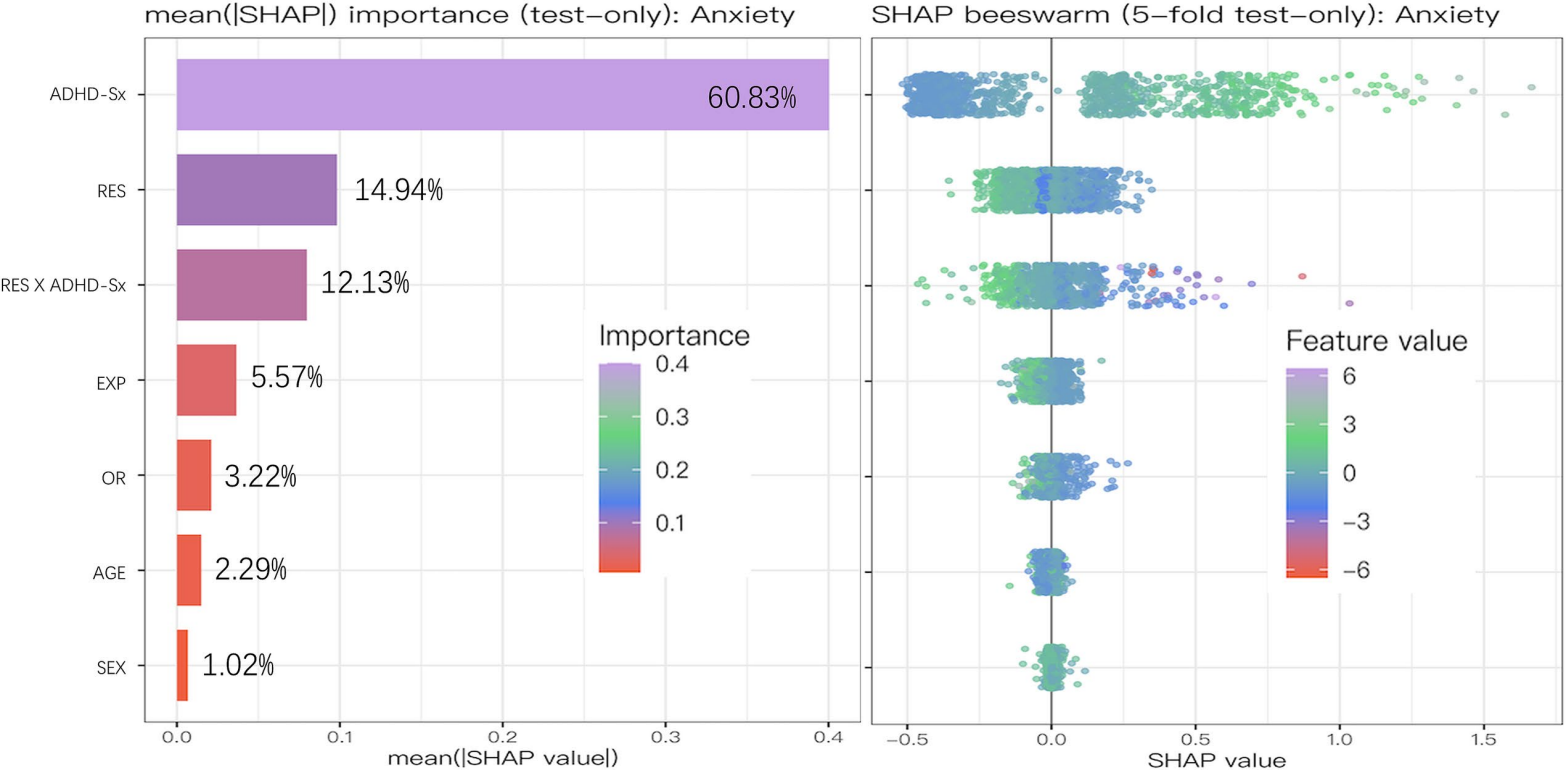
Feature importance and SHAP explanations for the XGBoost model predicting anxiety.

According to the model comparison results, SHAP-based interpretability analysis was conducted for the XGB model; and based on this, identify all contributing features by performing a key-predictor screening with this tool (as shown in Figures 2, 3). To ensure that the above-mentioned methods are not overly complex but can help readers more quickly understand the models’ internal working mechanisms after learning these approaches, which facilitates effective interpretation and use of the models. The results show that, for the ADHD-Sx feature extracted from the X-D component based on PCA (as described above), when predicting the scores of depression and anxiety, its mean absolute SHAP values were approximately 0.357 and 0.400, respectively, with contributions exceeding 56 per cent and 61 per cent. The next to the top features were psychological resilience (depression: 0.115, 18.07%; anxiety: 0.098, 14.94%), and the Resilience x ADHD-Sx interaction term (depression: 0.077, 12.06%; anxiety: 0.080, 12.13%). Together, these three theoretically relevant features accounted for the largest share of the aggregated SHAP-based feature importance, reaching 86.29% for depression prediction and 87.90% for anxiety prediction. This pattern indicates that ADHD-Sx, psychological resilience, and their interaction were the dominant contributors to the XGBoost model predictions. Orienteering participation, orienteering experience, age, and sex contributed to it less significantly. The beeswarm plots also showed that as the ADHD-Sx value increased, there was an increase in positive SHAP values; thus, predictions would lean toward higher levels of depression and anxiety. Conversely, when resilience values were high, there was a tendency for SHAP values to be negative, indicating a counter-acting effect of this factor on the prediction of internalizing symptoms. Resilience × ADHD-Sx interaction was one of the top-three features for both outcomes; There is a difference in the Systemic prediction effect on Resilience across symptoms and treatment outcome under different conditions of ADHD-Sx. This pattern is consistent with the direction of the moderated mediation and simple-slope results reported above, indicating a stronger negative association between resilience and depression/anxiety among individuals with higher ADHD-Sx.

## 4 Discussion

According to the association between orienteering and the internalization issue (depression, anxiety) of teenagers, this study will conduct statistical mediation testing and predictive verification to obtain results that generally conform. Orienteering was associated with a lower risk of depression and anxiety, but this the association was not mainly direct; instead, it was statistically reflected in resilience as a mediator. That is to say, orienteering can help reduce some negative feelings; However, it may also cause some anxiety through the transfer of an adaptive resource system. Second, Attention-Deficit/Hyperactivity Disorder symptoms (ADHD-Sx) significantly moderated this indirect association; although ADHD-Sx was associated with higher depression and anxiety, the protective effect of resilience on negative emotions became stronger as ADHD-Sx increased, making the indirect association of orienteering on internalizing problems through resilience more prominent among adolescents with higher ADHD-Sx. This pattern is a way to accumulate resources that brings mental health-related associations from cognitively engaged sports and defines the boundary conditions of this effect under a risk context, providing a new theoretical framework for understanding how cognitively challenging physical activities are related to adolescent mental health.

### 4.1 Resilience as an indirect association pathway

Based on the analysis of this study, it can be concluded that orienteering participation was positively associated with psychological resilience, and Resilience is significantly negatively correlated with depression and anxiety. After resilience was introduced as a factor, although there was still a direct association of orienteering on internalizing problems, this connection had become invalid due to the introduction of resilience as an intermediate variable; However, the indirect influence remained stable. This pattern aligns with the essential assumption of the Conservation of Resources theory that people seek to acquire, possess, and protect valuable resources, and the accumulation of resources can alleviate the stress caused by resource loss and help them successfully cope with adversity (Hobfoll et al., 2018). Orienteering, as a combined sport of physical load and continuous cognition-execution demands, offers a naturalistic setting for the construction of resilience-related resources. Under the simultaneous pressure of time and space, the participants need to continuously set goals, check positions, and monitor their own actions; if there is an error in route judgment or they are confused about the current situation, they should quickly recover orientation and adjust the strategy to continue performing the task. Baumeister and Vohs have proposed that the dynamic psychological cycle, including monitoring, adjustment, and reengagement, has an interpretation of this process of resources to preserve and restore for each individual at a microscopic level. Through the goal–feedback–adjustment cycle, individuals can enhance the cognitive flexibility, self-regulation, and emotion regulation components of resilience (Bonanno, 2004) to help develop and consolidate psychological resilience.

Notably, a key feature distinguishing orienteering from general physical activity is its high level of cognitive engagement. Compared with the simple physical activity oriented toward repetition that was prevalent earlier, Orienteering integrates cognitive assessment throughout its entire tasks; Actually, during an orienteering activity requires continuous thinking to maintain orientation information of surrounding environment; After getting map instruction, people must make judgments accordingly whether it is right or wrong direction change by reference, and then adjust course immediately if there are mistakes (Eccles et al., 2002). Prior work suggests that this “dual-task” structure may confer unique advantages for cognitive functioning and emotion regulation (Biehl-Printes et al., 2024; Waddington et al., 2024). Extending this line of reasoning to mental health, the present findings indicate that orienteering is linked to lower depression and anxiety via a resilience pathway, providing additional empirical support for the proposition that cognitively engaging forms of physical activity may be related to psychological health for psychological health. This finding is consistent with COR theory, which emphasizes that adaptive psychological resources can buffer individuals from stress-related emotional difficulties. In the context of orienteering, repeated navigation, decision-making, and error correction may provide opportunities for adolescents to mobilize and consolidate resilience-related resources.

### 4.2 Resource compensation under risk contexts: the moderating role of ADHD-Sx

Among the test results for boundary conditions, the most significant one was found to be the case. According to the above research results, we can know that: there is no significant change in the strength of the relationship between ADHD-Sx and orienteering participation and resilience; However, there is a strong moderation effect on resilience’s protective role against depression and anxiety. Specifically, when the score of ADHD-Sx increased, there was a significant increase in the negative correlation between resilience and negative emotions; And at this time, the indirect association of orienteering on internalizing problems through resilience became relatively stronger for adolescents with higher scores of ADHD-Sx.

This pattern can be understood through a risk–protective model or a resource-compensation perspective (Fergus and Zimmerman, 2005), which posits that the effects of protective factors tend to intensify as risk increases—under high-risk conditions, the marginal benefits of protective resources are more substantial. Adolescents with elevated ADHD-Sx generally face greater self-regulatory demands and a higher risk of emotional lability (Shaw et al., 2014), and difficulties in sustained attention and impulse control may leave them relatively resource-constrained in everyday coping contexts. In this context, resilience—reflecting the capacity to adapt to change, cope with stress, and regulate negative affect—becomes a more consequential protective resource. In other words, resilience may serve a more “compensatory” function among adolescents with higher ADHD-Sx, whereas in lower-risk groups its effect is comparatively smaller.

It is important to emphasize that this moderation pattern does not imply that ADHD-Sx is “beneficial.” On the contrary, ADHD-Sx was strongly and positively associated with both depression and anxiety in this study (*r* = 0.63 and 0.65), and it accounted for more than half of the predictive contribution in subsequent predictive models. This indicates that ADHD-Sx is a major risk factor for adolescent internalizing problems, and it is precisely under this elevated-risk context that the protective value of resilience becomes more fully expressed. From an intervention standpoint, these results imply that for adolescents with higher ADHD-Sx, resilience gains associated with orienteering may translate into larger emotional benefits, suggesting this subgroup may represent a high-yield target population for orienteering-based interventions.

### 4.3 Cross-validated machine-learning prediction

One important part of introducing the application of prediction models in this study is integrating the explanatory paradigm with the predictive paradigm to combine them. Traditionally, sport psychology research often operates in a hypothesis-verification system, while the present study treats machine learning methods as an alternative robustness test of the theory-based model to assess whether the essential conclusions are consistent among different analytic approaches. Based on these, it can be inferred that the three main directions for future research are as follows. First, the sufficiency of linear models supports the simplicity and efficiency of the theoretical specification. Both ridge regression and XGBoost showed high consistency in terms of fivefold cross-validation, and adding a more complicated non-linear model did not improve it significantly. Therefore, the predictive analyses offered a complementary perspective on the explanatory model. Across both outcomes, ADHD-Sx, psychological resilience, and their interaction consistently emerged as the most influential predictors in the XGBoost models. This convergence supports the relevance of these theoretically specified variables, while the moderate holdout performance also indicates that depressive and anxiety symptoms are not fully captured by the current feature set and may involve additional psychosocial, contextual, or biological factors. From a data-driven angle, the convergence also returns to theory building; When Theory-Driven and Data-Driven Models show consistency, the core conclusions have robust reliance and simple explanation. The second is that, based on cross-modal agreement, the degree of support for hypotheses may provide additional evidence for the moderating effect of variables. In terms of prediction, the Shapley values independently reproduced the main pattern: ADHMSx + Psychological resilience + their interactions explained more than 86% of the total explanatory power across the globe; The direction of this contribution was consistent with the moderated theory that suggests resilience has a stronger protective effect on high-ADHMSx adolescents. Across different approaches, such an agreement increases trust in the results’ level of “statistical significance” and “reproducibility,” effectively alleviating current doubts about the reproducibility problem in psychology. Secondly, the explanation of the Area of the prediction model also shows a clear Direction for further Research. The rest of the unexplained variance suggests that, while the current features capture most of the mechanisms underlying adolescents’ internalizing behaviors, there may be other factors at multiple levels affecting these problems (for example, social support and physiological variables) that are yet to be covered in the existing research, thus opening up a way forward for both theory and practice exploration.

### 4.4 Practical implications

The above results also have practical applications in school-based physical education and interventions for adolescent mental health. Since the link between orienteering and internalizing problems seems to operate mainly through psychological resilience, dissemination efforts should take psychological resilience as a central objective and help students accumulate transferable psychological resources through repeated cycles of “setback-adjustment-re-engagement.” In addition, adolescents with higher ADHD-Sx may benefit more; therefore, providing a more supportive task structure for this subgroup (such as staged goal setting and timely feedback mechanisms) may enhance the efficiency at which resilience gains translate into emotional benefits. Finally, as an “oriented task” that integrates physical load and high cognitive demands, it may also bring about psychological benefits in addition to those brought about by activities focusing mainly on repetitive physical output. Such cognitively engaging sports can therefore be used as a diverse and ecologically valid vehicle for nurturing resilience among middle school students.

### 4.5 Limitations and future directions

When interpreting the results and planning future research, several limitations should be considered. First, the cross-sectional design restricts causal inference; orienteering participation and resilience may be bidirectionally related. Longitudinal design and randomized controlled intervention trials are required to verify the temporal order and cause-and-effect relationship of resilience accumulation. Secondly, since the sample was mainly drawn from Sichuan, China, generalization to other cultural backgrounds should be done with caution. In addition, participation in orienteering was evaluated through self-report, which is prone to recall bias and social desirability effects; In the future, objective indicators (such as GPS-based movement tracks, heart rate monitoring) and multi-informant reports could be introduced to enhance the validity of measurement. Third, although the predictive analysis suggests that the current feature set has captured most of the predictive signals, other potential moderators (such as social support and family environment) have not been included and should be considered in future research. Finally, ADHD-Sx was defined as a continuous symptom dimension, in line with the dimensional approach; however, care should be exercised when generalizing the results to clinically diagnosed ADHD populations. In the future, both the clinical group and the non-clinical group need to be added to the research to explore whether there is a general moderation effect across all levels.

## 5 Conclusion

Orienteering participation is significantly related to lower depressive and anxious emotions in adolescents, and this association was statistically reflected in indirect associations via psychological resilience; new empirical evidence has been provided for the conservation of resources theory. ADHD-Sx significantly moderated the association of resilience; that is, when the symptom level is high, the negative correlation between resilience and negative emotions is more pronounced, and orienting is more likely to have a positive association on depression or anxiety through resilience. The predictive analyses further showed that ADHD-Sx, psychological resilience, and their interaction were consistently important contributors to model predictions, providing supplementary evidence for the relevance of these variables in adolescent internalizing problems. In summary, the overall results indicate that resilience-based pathways and their boundary conditions connecting orienteering with adolescent internalizing problems have been established; thus, it is proposed that school-based mental health intervention programs should enhance resilience-building and provide more supportive task structures for adolescents with higher levels of ADHD-Sx.

## Data Availability

The raw data supporting the conclusions of this article will be made available by the authors, without undue reservation.
